# Morphology and composition controlled synthesis of flower-like silver nanostructures

**DOI:** 10.1186/1556-276X-9-302

**Published:** 2014-06-14

**Authors:** Ning Zhou, Dongsheng Li, Deren Yang

**Affiliations:** 1 State Key Laboratory of Silicon Materials and Department of Materials Science and Engineering, Zhejiang University, Hangzhou 310027, People's Republic of China; 2 Cyrus Tang Center for Sensor Materials and Applications, Zhejiang University, Hangzhou 310027, People's Republic of China

**Keywords:** Flower-like, Silver nanostructure, Hexagonal close-packed, Overgrowth, SERS

## Abstract

Flower-like silver nanostructures with controlled morphology and composition were prepared through wet-chemical synthesis. The reaction rate is simply manipulated by the amount of catalyzing agent ammonia added which is the key point to determine the ratio of hexagonal close-packed (HCP) to face-centered cubic (FCC) phase in silver nanostructures. The existence of formic acid that is the oxidation product of aldehyde group is demonstrated to play a crucial role in achieving the metastable HCP crystal structures by replacing ionic surfactants with polyvinylpyrrolidone (PVP). Utilizing flower-like silver nanostructures as surface-enhanced Raman scattering (SERS) substrates, Raman signal of Rhodamine 6G, or 4-aminothiophenol with concentration as low as 10^−7^ M was detected. Moreover, it is demonstrated that phase composition has no direct relation to the SERS enhancing factor which is mainly determined by the amount of hot spots.

## Background

In the last decades, it has been demonstrated that metallic nanostructures are a powerful means to attain the subwavelength control of electromagnetic field thanks to the so-called surface plasmon (SP) effect supported by them [1,2]. Confining the oscillating collective excitations at the interface of a metal and a dielectric introduces the prospect of optical devices with new functionalities by enhancing inherently weak physical processes, such as fluorescence [3] and Raman scattering which the latter is nominally called surface-enhanced Raman scattering (SERS) [4].

Surface plasmon and electrooptical properties can be effectively and intentionally regulated by the size and shape of the nanostructure. Various morphology-controlled noble metal structures have been synthesized among which flower-like silver nanostructures raise much attention and are promising candidates as SERS substrate owing to silver-intrinsic outstanding properties than other metals [5], the existence of abundance of ‘hot spots’ in sharp tips and nanoparticle junctions resembling intuitively nanoscale optical antenna [6,7].

Nowadays, many approaches including chemical reduction [8,9], light irradiation [7], galvanic replacement [10], evaporation [11], and anisotropic etching [12] have been developed to prepare flower-like noble metal nanostructures. Metal nanostructures with well-controlled shape, size, and uniquely designed optical properties can be finely prepared with multistep methods such as double-reductant method, etching technique, and construction of core-shell nanostructures [13]. In comparison, although single-step reduction needs to be regulated carefully and improved intentionally, this method can be more efficient.

In the solution-phase synthesis, nanocrystals of common face-centered cubic (FCC) metals tend to take a polyhedral shape [14]; therefore, highly branched Ag nanostructures are thermodynamically unfavorable. In our previous research, flower-like silver nanostructures were synthesized employing CH_2_O or C_2_H_4_O as a moderate-reducing agent [15,16]. The reaction is finished in less than 1 min; thus, the growth rate is beyond the thermodynamically controlled regime, which leads to anisotropic growth due to a faster rate of atomic addition than that of adatom diffusion.

However, kinetic-controlled growth alone cannot interpret the occurrence of unusual and rare hexagonal close-packed (HCP) silver nanostructures apart from common FCC ones as noted in our previous report [15]. To our knowledge, HCP crystal structures appear in silver nanowires prepared by electrochemical deposition [17-19] or by simply heating or evaporating FCC-Ag nanowires or nanoparticles [20,21]. Various metal nanostructures containing HCP structures with different morphologies including Ag belts [22], prisms [23], needles [24], rices [25], Au square sheets [26], and tadpoles [27] have been researched. As to crystal structure composition, except the researches [18,26] in which the composition are exclusively HCP, HCP coexists with FCC in most of the aforementioned reports. Ag nanowires with diameters around 30 nm prepared by electrochemical deposition are found to have the highest concentration in the total of HCP to FCC nanowires [17]. However, there are few reports about regulating the ratio of HCP to FCC in solution-phase synthesis and further researching the reaction parameters affecting it, neither the inherent growth mechanism.

In this paper, the size and morphology of the flower-like silver nanostructures and further the ratio of HCP to FCC phase can be manipulated by varying the amount of catalyzing agent added to the solution. Considering there exists an optimal point where HCP phase is the richest together with the indispensable factor of the nature of stabilizing agents, the proposed growth mechanisms is corroborated. Utilizing these flower-like Ag nanostructures as SERS substrates, the Raman signal of Rhodamine 6G (R6G) or 4-aminothiophenol (4-ATP) with concentration 10^−7^ M can be recognized due to numerous hot spots.

## Methods

Aqueous solution (37% CH_2_O, 28% NH_3_•3H_2_O, and 40% C_2_H_4_O) was purchased from Sinopharm Chemical Reagent Co. Ltd (Shanghai, China). Polyvinylpyrrolidone (PVP, k30), AgNO_3_, sodium sulfate (SS), and sodium dodecyl sulfate (SDS) with analytical pure grade were supplied by the same corporation. R6G (98%) and 4-ATP (97%) was purchased from Sigma-Aldrich Company (Shanghai, China).

In a typical synthetic procedure, 200 mL 0.25 mM AgNO_3_ aqueous solution at 45°C was sequentially added to 0.1 mL aqueous solution of 37% CH_2_O and 0.4 mL 28% NH_3_•3H_2_O. It is worth mentioning that NH_3_•3H_2_O should be injected rapidly. After 1 min, 10 mL 10% (*w*/*w*) PVP aqueous solution was mixed into the solution so as to stabilize the silver nanostructures. After 4 more min, the product was collected by centrifugation at 6,000 r min^−1^. The amount of NH_3_•3H_2_O varied from 200 to 800 μL, and for simplification, the silver nanostructures samples are denoted as P200, P400, P600, and P800, respectively. To verify the directing role of formic acid, which is the oxidation product of CH_2_O, SS or SDS instead of PVP was injected in similar concentration and the silver nanostructures samples are denoted as SS400 and SDS 400, respectively.

The morphology of the samples was characterized by a scanning electron microscope (SEM, Hitachi S-4800). The phase constitution of the samples was examined by X-ray diffraction (XRD) using an X'Pert PRO X-ray diffractometer equipped with the graphite monochromatized Cu Kα radiation. The extinction spectra of the samples were measured on Ocean Optics spectrophotometer with an optical path of 10 mm over the range of 200 to 1,100 nm. The integration time is 6 ms.

To employ flower-like Ag NPs as SERS substrate, firstly, the flower-like particles were deposited onto a square silicon wafer with side length of 10 mm, and then immersed in 10^−7^ M ethanol solution of R6G or 4-ATP for 6 h. Bare silicon wafers were also immersed in 10^−2^ M R6G or 4-ATP solution for comparison. After thoroughly rinsed with ethanol and drying by nitrogen, they were subjected to Raman characterization. The data were obtained by choosing six different spots of the sample to average. The SERS spectra were recorded using a Bruker SENTERRA confocal Raman spectrometer coupled to a microscope with a × 20 objective (N.A. = 0.4) in a backscattering configuration. The 532-nm wavelength was used with a holographic notch filter based on a grating of 1,200 lines mm^−1^ and spectral resolution of 3 cm^−1^. The Raman signals were collected on a thermoelectrically cooled (−60°C) CCD detector through 50 × 1,000 μm × 2 slit-type apertures. SERS data was collected with laser power of 2 mW, a laser spot size of approximately 2 μm, and integration time of 2 s. The Raman band of a silicon wafer at 520 cm^−1^ was used to calibrate the spectrometer.

## Results and discussion

**Figure 1:**
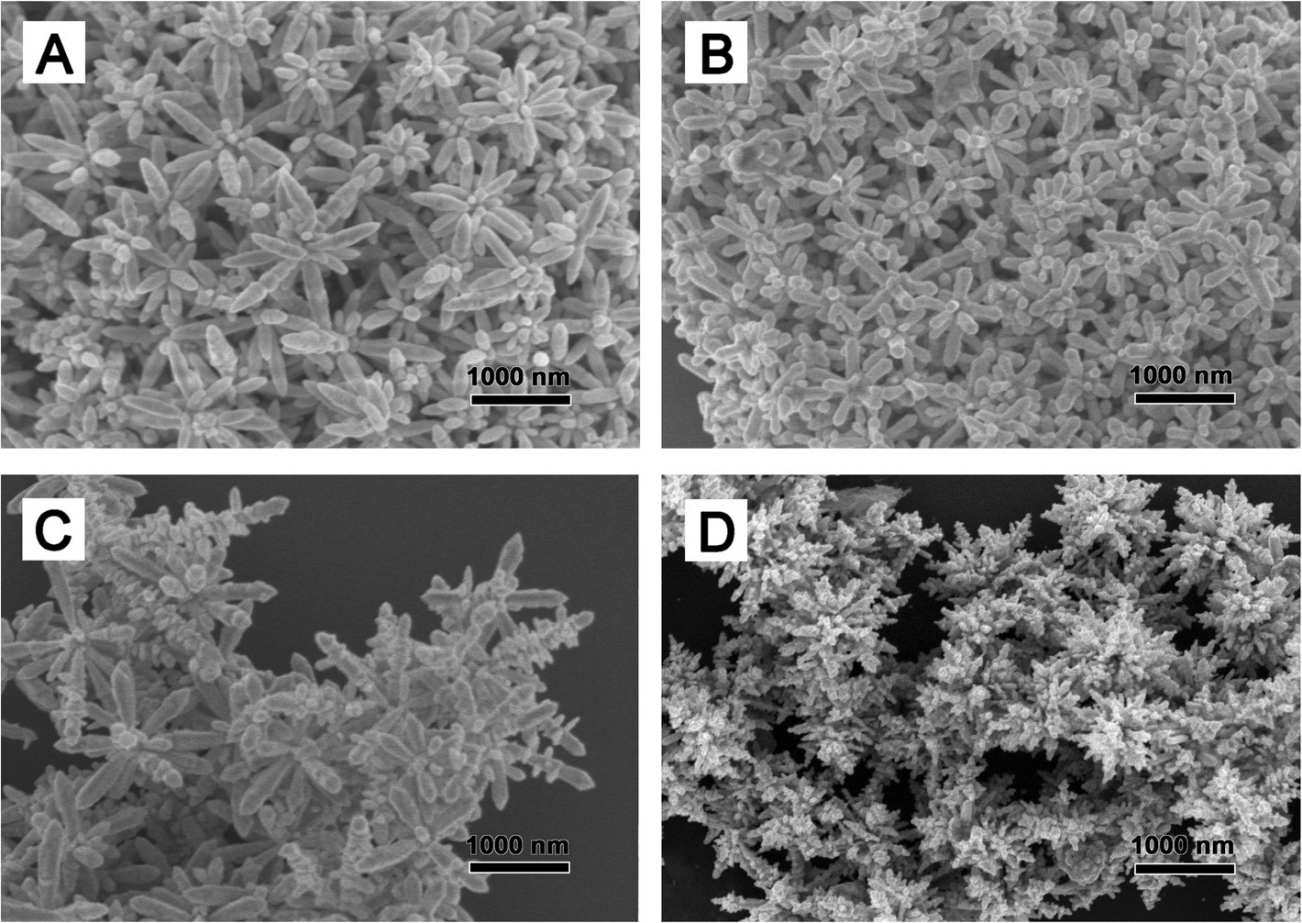
**SEM images of the flower-like Ag nanostructures.** SEM images of the flower-like Ag nanostructures prepared with PVP and different amounts of catalyzing agent NH_3_•3H_2_O: **(A)** 200 μL, **(B)** 400 μL, **(C)** 600 μL, and **(D)** 800 μL.

The SEM images of the flower-like Ag nanostructures with different amounts of catalyzing agent NH_3_•3H_2_O are shown in Figure 1. All the flower-like Ag nanostructures consisting of a silver core and many rod-like tips protruding out are abundant with higher curvature surface such as tips and sharp edges compared to the highly branched nanostructures in previous reports [28,29]. There is a trend that the constituent rods become smaller in both longitudinal dimension (from about 1 μm to dozens of nanometers) and diameter (from 150 nm to less than 50 nm) as the amount of catalyzing agent NH_3_•3H_2_O increases. Meanwhile, the rods become abundant; consequently, the junctions or gaps between two or more closely spaced rods turn to be rich. One interesting thing deserving to be mentioned is that there is a turning point in which various kinds of rods with different length and diameters coexist when the amount of NH_3_•3H_2_O is 600 μL (Sample P600) as shown in Figure 1C .

**Figure 2:**
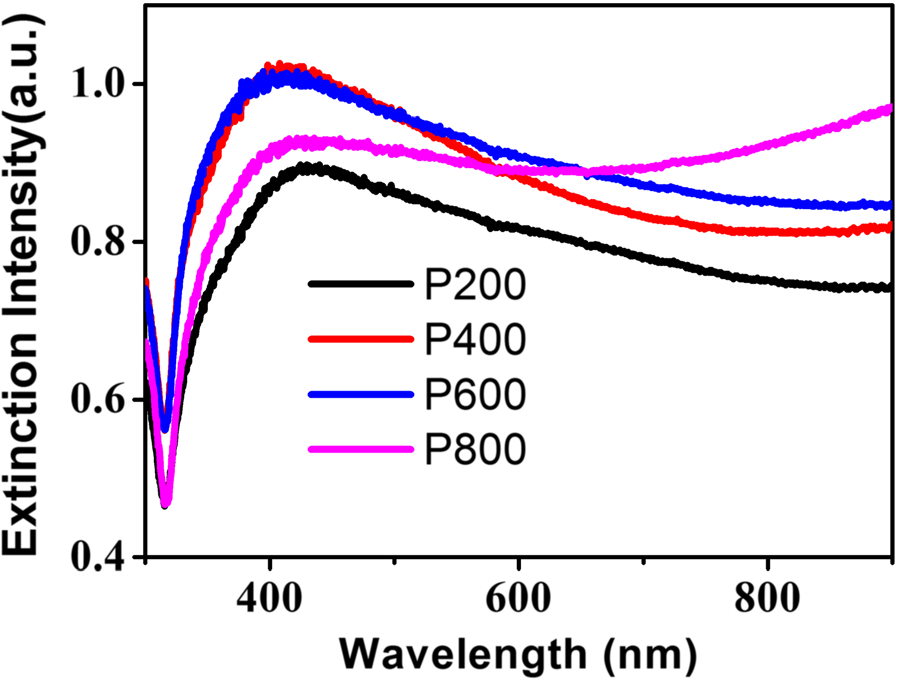
**The extinction spectra of the flower-like Ag nanostructures.** The extinction spectra of the flower-like Ag nanostructures prepared with PVP and different amounts of catalyzing agent NH_3_•3H_2_O. In the legend of the figure, for simplification, the samples are denoted as P200, P400, P600, and P800, respectively. ‘P’ stands for ‘PVP’ and the followed number stands for the volume of NH_3_•3H_2_O added.

In solution-phase synthesis of highly branched noble metal nanostructures, the reaction rate and the final morphology can be manipulated by the concentration of the precursor [30], the reaction time [9], the trace amount of salts such as Cu^2+^, Fe^2+^, or Fe^3+^[31], and so on. In the case of our synthesis, the reaction rate is dominated by the amount of catalyzing agent NH_3_•3H_2_O injected. As ammonia is added, the pH value of the solution is raised leading to initiation of Ag^+^ reduction to Ag^0^ atoms. Meanwhile, because there is no capping agent in the solution, the solution is supersaturated with Ag^0^ atoms after reduction reaction and primary small particles form through a burst-nucleation step. At the growth stage, large particles form with different morphologies and sizes through diffusional growth or aggregation. The reaction is finished in less than 1 min, and these two stages are tough to be distinguished separately and potentially take place at the same time. So, the growth rate is in the kinetic-controlled regime, which is classified as kinetically controlled overgrowth in a minireview [14]. Anisotropic overgrowth occurs due to a faster rate of atomic addition or small particles aggregation than that of adatom diffusion, with high-energy facets growing more quickly than low-energy facets; hence, fast growth rate is indispensable to appearance of flower morphology. Larger quantity of ammonia leads to more fast reaction rate and more Ag^0^ atoms forming at initial stage. Consequently, the adatoms and small particles have less time to diffuse or aggregate. Compared to sample P400 denoting 400 μL NH_3_•3H_2_O injected, in P600 reaction condition, more adatoms burst as soon as NH_3_•3H_2_O is added; high growth rates occur at areas with high curvature of the rods; and secondary branches begin to grow from the main branches. This can explain the appearance of aforementioned turning point displayed in Figure 1C. Further increasing the NH_3_•3H_2_O addition, there is an insufficient supply of silver atoms to support the growth stage giving rise to flower cluster formation with abundant rods but limited rod length in Figure 1D. P200 has more time to diffuse and forms large rods with the length as long as 1 μm. This is well displayed in the extinction spectra (Figure 2) in which the surface plasmon resonance peak is red shift compared to others although they all exhibit broad spectra from visible to near-infrared range due to complex morphology and hybridization of plasmons associated with longitudinal plasmon resonance of rods and multipole resonance. With increasing the amount of NH_3_•3H_2_O, less diffusion time leads to short rods and the main surface plasmon resonance peak is slightly blue shift and the full width at half maximum becomes larger. When it comes to 800 μL, there is a lifting in near-infrared region probably because flower clusters with abundant rods form as displayed in Figure 1D and multipole resonance becomes dominant.

**Figure 3:**
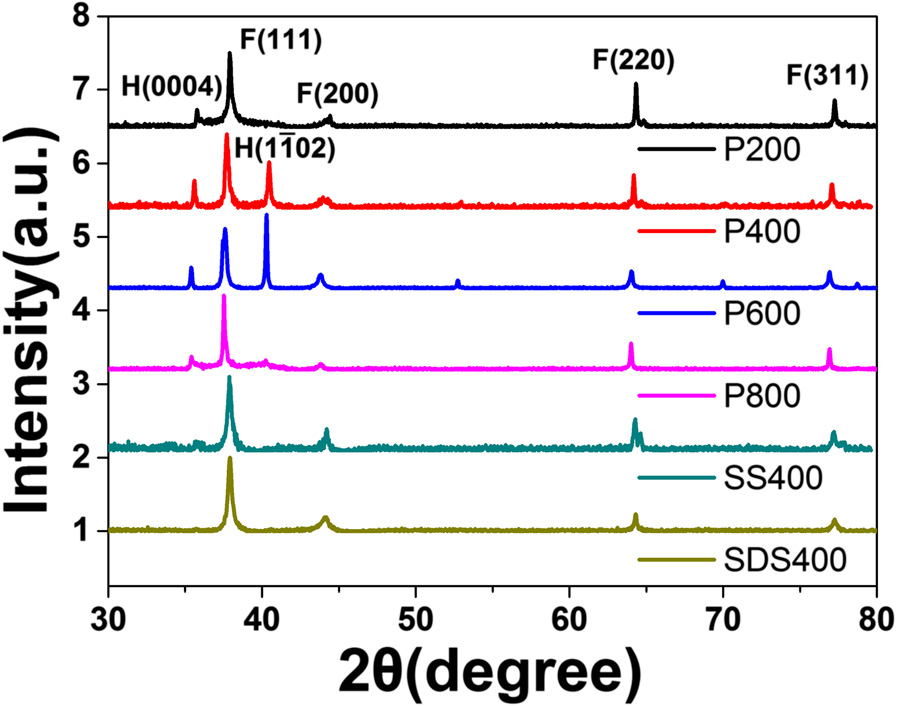
**The XRD spectra of different flower-like Ag nanostructures.** The XRD spectra of different flower-like Ag nanostructures prepared with different stabilizing agents and different amounts of catalyzing agent NH_3_•3H_2_O. In the legend of the figure, ‘P’ stands for PVP, ‘SS’ stands for sodium sulfate, ‘SDS’ stands for sodium dodecyl sulfate, and the followed number stands for the amount of NH_3_•3H_2_O added.

The crystal structure of the samples was characterized by XRD as presented in Figure 3. Different peaks corresponding to different plans have been marked. Obviously, FCC structures exist in all the samples. Apart from common FCC structures, the unusual and rare HCP silver nanostructures appear in all the samples stabilized by PVP. Further analysis demonstrates that there is a point in which the ratio of HCP to FCC phase is highest when the amount of NH_3_•3H_2_O is 600 μL which coincidently corresponds to morphology turning point. Before this point, the ratio of HCP to FCC phase increases, and after that, the trend is contrary. Thus, the amount of HCP phase does not change linearly with the number of rods as displayed in Figure 1. Fast reaction is not very important for the appearance of HCP phase as noted in our previous report [15], but very essential for the growth of rod-like tips. In this paper, we demonstrate that reaction rate is the dominant factor influencing the ratio of HCP to FCC phase, namely, the abundance of HCP in silver nanostructures. However, another question arises what is the dominated factor for the abundance of HCP.

HCP Ag structures have a more favorable surface configuration but higher volume internal energy than FCC Ag. Common bulk silver is well known as a FCC metal because FCC Ag has a lower internal energy when surface and interface effect can be neglected. However, when it comes to nanometer dimension, the surface energy may play a major role in determining the crystal structure and must be taken into consideration. Thus, the metastable HCP phase can have a more stable surface configuration at a certain shape and size range [17,24,25]. By using electrochemical deposition, HCP structural silver nanowire is discovered to coexist with FCC one and the highest concentration of HCP-Ag nanowire appears when the diameters are around 30 nm [17]. As for our preparation, with increasing the amount of catalyzing agent NH_3_•3H_2_O, the protruding rods become smaller in both longitudinal dimension and diameter as mentioned above. Smaller rods are occupied by larger surface areas, so HCP Ag structures become more favorable resulting in highest ratio of HCP to FCC phase when the amount of NH_3_•3H_2_O is 600 μL. Further increasing the amount of NH_3_•3H_2_O leads to numerous rods assembled in Ag clusters (Figure 1D), which may be the reason for the reduction of HCP percentage.

**Figure 4:**
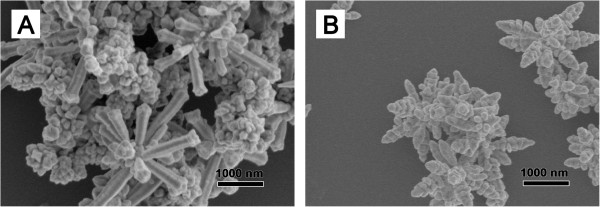
**SEM images of the samples stabilized by ionic surfactants.** SEM images of the samples stabilized by **(A)** SS and **(B)** SDS.

Except the effect of the morphology, the growth mechanism/conditions as well play an important role in achieving the metastable high-energy crystal structures in nanometer-scale systems [18]. In our experiment, carboxyl group (-COOH) which is the oxidation product of aldehyde group may be beneficial for the formation of HCP phase [11,15]. To demonstrate this, SS or SDS instead of PVP was injected in a similar concentration. Figure 4 indicates that the products are both flower like except that the rods are more coarse and larger in transverse dimension. However, there is no HCP phase in both samples as displayed in Figure 3. This phenomenon can be interpreted that PVP as a kind of polymer surfactants has no effect on the oxidation product of CH_2_O. Contrarily, SS or SDS can disturb the directing role of formic acid as both of them are ionic surfactants. Thus, formic acid is the essential factor in the existence of HCP phase.

**Figure 5:**
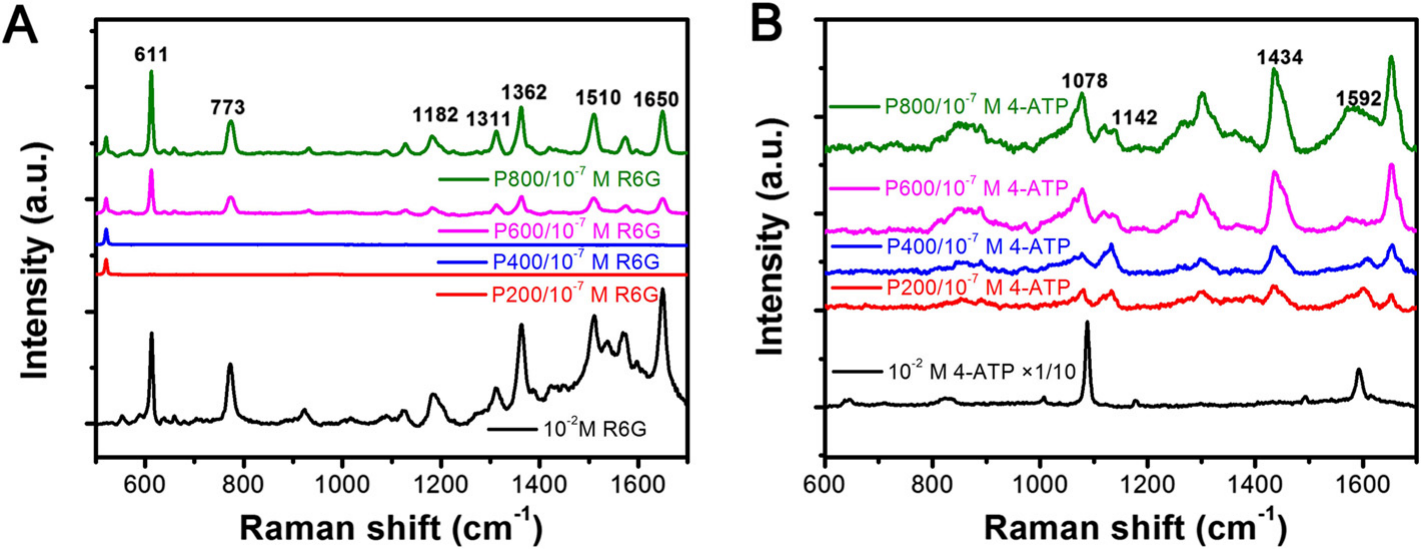
**SERS spectra and Raman Spectra of R6G and 4-ATP.** SERS spectra of 10^−7^ M R6G **(A)** and 4-ATP **(B)** using flower-like Ag nanostructures as SERS substrates, and Raman spectra 10^−2^ M R6G and 4-ATP on bare silicon wafer are also presented for comparison.

Utilizing flower-like Ag nanostructures as SERS substrate, the Raman signal of R6G as low as 10^−7^ M can be recognized in Figure 5A when P600 and P800 were used. This is not the case for P200 and P400. Different samples have different amounts of hot spots which reside in two types of areas, one is the high curvature surface in tips and sharp edges of rods, and the other is junctions or gaps between two or more closely spaced rods. Unlike P200 and P400, P600 is rich in secondary branches growing from main branches. P800 resembles flower clusters with abundant rods, and the hot spots should be the richest [6]. We further use 4-ATP as Raman active probe because of its strong chemical affinity to Ag and the large SERS signal. Compared to the spectrum obtained in pure 4-ATP, the SERS spectrum exhibits some distinct frequency shifts as displayed in Figure 5B because the -SH group of 4-ATP directly contacts with the Ag nanostructures surface by forming a strong Ag-S bond [32]. The bands at 1,592 and 1,078 cm^−1^ are attributed to the a_1_ modes of the 4-ATP molecule, and the bands at 1,434 and 1,142 cm^−1^ are assigned to the b_2_ modes [33]. As in the case of R6G as Raman active probe, the SERS intensity is maximum when P800 is used indicating that the electric field enhancement is the dominant factor for SERS in our samples. It is worthy to note than the Raman signal of 4-ATP as low as 10^−7^ M can be recognized in all the samples perhaps due to strong chemical affinity to Ag and the large SERS signal of 4-ATP compared to R6G molecules.

The different optimal parameters for SERS enhancement and HCP phase content indicate that the SERS enhancement factor has no direct relation with phase composition. As is well known, different crystal structures correspond to different spacial stacking of atoms. The HCP structure corresponds to the ABA sequence, whereas with FCC, the sequence is ABC [21]; thus, different crystal structures mean different carrier concentration and further plasma frequency [34]. Moreover, it has been demonstrated that SERS intensity strongly depends on the surface crystallographic orientation [35]. However, SERS detection in our characterization employed far-field Raman microscope which characterizes an electromagnetic field-average effect [36,37], and the lighting effect in the flower-like nanostructures with huge amount of sharp tips may overwhelm the crystal facet effect. Consequently, the influence of phase difference cannot be directly reflected in Raman spectra.

## Conclusions

In this paper, the size and ratio of HCP to FCC phase in synthesized flower-like Ag nanostructures are well controlled by tuning the amount of catalyzing agent ammonia added to the solution. There indeed exists an optimal point where HCP is the richest. Ionic surfactants may have an adverse effect on the formation of HCP phase through its influence on the oxidation product of aldehyde group. The flower-like Ag NPs can be employed as SERS substrate, and the SERS enhancement factor is related to amounts of hot spots and has no direct relation with phase composition.

## Abbreviations

HCP: hexagonal close-packed; FCC: face-centered cubic; SPs: surface plasmons; SERS: surface-enhanced Raman scattering; SEM: scanning electron microscope; XRD: X-ray diffraction; PVP: polyvinylpyrrolidone; SS: sodium sulfate; SDS: sodium dodecyl sulfate; R6G: Rhodamine 6G; 4-ATP: 4-aminothiophenol.

## Competing interests

The authors declare that they have no competing interests.

## Authors’ contributions

NZ performed the experiments, collected and analyzed the data, and wrote the paper; DL conceived the experiments, analyzed the results, and wrote the paper; DY helped with the data analysis and wrote the paper. All authors read and approved the final manuscript.
